# Whole-Brain Monosynaptic Inputs to Hypoglossal Motor Neurons in Mice

**DOI:** 10.1007/s12264-020-00468-9

**Published:** 2020-02-24

**Authors:** Han Guo, Xiang-Shan Yuan, Ji-Chuan Zhou, Hui Chen, Shan-Qun Li, Wei-Min Qu, Zhi-Li Huang

**Affiliations:** 1 Department of Pharmacology, School of Basic Medical Sciences; State Key Laboratory of Medical Neurobiology and Ministry of Education Frontiers Center for Brain Science, Institutes of Brain Science, Fudan University, Shanghai, 200032 China; 2 Department of Anatomy, School of Basic Medical Sciences, Fudan University, Shanghai, 200032 China; 3 Department of Pulmonary Medicine, Zhongshan Hospital, Fudan University, Shanghai, 200032 China

**Keywords:** Hypoglossal motor neuron, Monosynaptic input, Rabies virus, Respiration, Sleep and wake

## Abstract

Hypoglossal motor neurons (HMNs) innervate tongue muscles and play key roles in a variety of physiological functions, including swallowing, mastication, suckling, vocalization, and respiration. Dysfunction of HMNs is associated with several diseases, such as obstructive sleep apnea (OSA) and sudden infant death syndrome. OSA is a serious breathing disorder associated with the activity of HMNs during different sleep–wake states. Identifying the neural mechanisms by which the state-dependent activities of HMNs are controlled may be helpful in providing a theoretical basis for effective therapy for OSA. However, the presynaptic partners governing the activity of HMNs remain to be elucidated. In the present study, we used a cell-type-specific retrograde tracing system based on a modified rabies virus along with a Cre/loxP gene-expression strategy to map the whole-brain monosynaptic inputs to HMNs in mice. We identified 53 nuclei targeting HMNs from six brain regions: the amygdala, hypothalamus, midbrain, pons, medulla, and cerebellum. We discovered that GABAergic neurons in the central amygdaloid nucleus, as well as calretinin neurons in the parasubthalamic nucleus, sent monosynaptic projections to HMNs. In addition, HMNs received direct inputs from several regions associated with respiration, such as the pre-Botzinger complex, parabrachial nucleus, nucleus of the solitary tract, and hypothalamus. Some regions engaged in sleep–wake regulation (the parafacial zone, parabrachial nucleus, ventral medulla, sublaterodorsal tegmental nucleus, dorsal raphe nucleus, periaqueductal gray, and hypothalamus) also provided primary inputs to HMNs. These results contribute to further elucidating the neural circuits underlying disorders caused by the dysfunction of HMNs.

**Electronic supplementary material:**

The online version of this article (10.1007/s12264-020-00468-9) contains supplementary material, which is available to authorized users.

## Introduction

The hypoglossal nucleus (12N) is located in the dorsomedial medulla oblongata and consists of cholinergic hypoglossal motor neurons (HMNs). HMNs innervate several extrinsic and intrinsic tongue muscles that participate in basic and important motor functions, including swallowing, mastication, suckling, vocalization, and respiration [1–5]. Dysfunction of HMNs leads to physiological behavioral disorders and is associated with several diseases, such as obstructive sleep apnea (OSA) and sudden infant death syndrome [6]. OSA is a serious breathing disorder caused by sleep-dependent changes in neuromodulators acting on critical pharyngeal motor pools. Among these pools, HMNs innervating the genioglossus (GG) have been explored most extensively, as the GG is the largest and most accessible upper airway dilator [7]. Aberrant changes in the activity of HMNs have been demonstrated to induce low muscular tone of the GG and represent the key pathogenesis of OSA. A recent study in rodents demonstrated that chemogenetic activation of HMNs causes significant and sustained increases in GG activity during sleep but without effects on sleep–wake states [8], indicating that modulation of HMNs may be a potential means of improving ventilation during sleep in OSA. The activity of HMNs is modulated by excitatory and inhibitory signals in the brain during physiological behaviors [9–11]. However, it is not clear which specific excitatory or inhibitory signals directly control HMNs. Therefore, identifying the whole-brain inputs to HMNs is critical for a better understanding of the modulation of HMN activity during different behaviors.

Conventional approaches with non-specific tracers have been used to classify the major inputs from the brainstem to the 12N [12–14], while little attention has been paid to whole-brain inputs. Using horseradish peroxidase, a non-specific tracer, a previous study showed that the 12N mainly receives inputs from brainstem reticular regions, the nucleus of the solitary tract (Sol), and the sensory trigeminal complex [14]. Due to their poor cell-type specificity, non-specific tracers easily exceed the range of the 12N and spread to other nuclei, which has led to inaccurate results [14]. Pseudorabies virus has been injected into tongue muscles to explore the multisynaptic afferents to 12N; although this method improves efficiency, it cannot accurately identify monosynaptic transmission [12, 15]. Therefore, it is essential to adopt a cutting-edge viral tracing system with high specificity to identify monosynaptic inputs that target the 12N to yield a comprehensive anatomical-functional understanding of HMNs.

To avoid the limitations of traditional technology, we combined a genetically modified rabies virus for trans-synaptic retrograde tracing [16] with the Cre/loxP gene expression system [17] to comprehensively identify the monosynaptic inputs onto HMNs from the entire brain.

## Materials and Methods

### Animals

Adult choline acetyltransferase (ChAT)-IRES-Cre mice [18] of the C57BL/6J strain and non-Cre-expressing littermates (2–4 months old) were used for retrograde tracing experiments. Adult GAD2-IRES-Cre mice [19] and calretinin (CR)-IRES-Cre mice were used for anterograde tracing experiments. Mice were bred and housed under a 12/12 light/dark cycle (lights on at 07:00) at 22 °C ± 1 °C with 55% ± 5% humidity, and were provided with unlimited food and water [20]. All experiments were approved by the Committee on the Ethics of Animal Experiments of the Basic Medical Sciences School at Fudan University (permit number 20140226-024).

### Viruses and Surgery

AAV2/9-CAG-DIO-TVA-GFP (1.7 × 10^13^ genome copies/mL), AAV2/9-CAG-DIO-RG (6.8 × 10^12^ genome copies/mL), and an EnvA-pseudotyped glycoprotein (RG)-deleted and DsRed-expressing rabies virus (RV-EnvA-ΔG-DsRed, 5.0 × 10^8^ fluorescence-forming units/mL) were purchased from BrainVTA (Wuhan, China) [21, 22]. The two Cre-dependent AAVs were mixed at a 1:1 ratio in 100 nL as helper viruses for retrograde monosynaptic-tracing experiments. AAV-EF1α-DIO-ChR2-mCherry (3 × 10^12^ genome copies/mL) was used for anterograde tracing experiments and was provided by Taitool Bioscience Co., Ltd (Shanghai, China).

Surgical procedures were performed as described in previous studies [22, 23]. Briefly, mice were anesthetized with chloral hydrate (350 mg/kg, intraperitoneal) and placed in a stereotaxic apparatus. A glass micropipette was used to deliver a viral vector to the 12N (antero-posterior [AP], − 7.5 mm; medio-lateral [ML], − 0.3 mm; dorso-ventral [DV], 4.5 mm) after exposing the skull. The viral vector was microinjected with a compressed-air delivery system, as described previously [24]. For retrograde tracing, helper viruses (100 nL) were injected into the 12N and left in place for 10 min to allow their diffusion away from the injection site. Fourteen days later, 200 nL of RV-EnvA-ΔG-DsRed was injected into the same location. After one week, these mice were perfused for immunostaining (*n* = 4). For anterograde tracing, AAV-EF1α-DIO-ChR2-mCherry was injected into the central amygdaloid nucleus (CeA; AP, − 1.22 mm; ML − 2.4 mm; DV, 4.8 mm) of GAD2-Cre mice and into the parasubthalamic nucleus (PSTN; AP, − 2.0 mm; ML, − 1.1 mm; DV, 5.0 mm) of CR-Cre mice, using the procedures described above. After three weeks, all of these mice were perfused.

### Histology and Immunostaining

Mice were perfused with 50 mL PBS, followed by 100 mL 4% paraformaldehyde in PBS. Brains were removed, post-fixed for 24 h at 4 °C, and then cryoprotected in 30% sucrose until they sank. Sections were cut at 30 μm on a freezing cryostat (CM1950; Leica, Wetzlar, Germany).

To confirm that the neurons initially infected were cholinergic neurons in the 12N, we immunostained sections containing the 12N with primary antibodies to ChAT. To characterize the inputs and initially-infected neurons of the CeA and PSTN, we immunostained sections containing these areas with primary antibodies against γ-aminobutyric acid (GABA) for the CeA and CR for the PSTN [21, 22]. The sections were incubated overnight at 4 °C in PBST (PBS with 0.3% Triton X-100 [*v*/*v*]) and with the following primary antibodies: goat anti-ChAT (1:500, cat. #AB144P, Millipore, Billerica, MA), rabbit anti-GABA (1:1000, cat. #PA5-32241, Life Technologies, Carlsbad, CA), and rabbit anti-CR (1:2000, cat. #7697, Swant, Bellinzona, Ticino, Switzerland). After three washes in PBS, the sections were incubated with an Alexa Fluor-conjugated IgG antibody (1:1000, Invitrogen, Carlsbad, CA) at room temperature (RT, 20 °C–22 °C) for 2 h. Then, the sections were counterstained with 4′, 6-diamidino-2-phenylindole (DAPI, 1:3000, cat. # D9542, Sigma-Aldrich, St. Louis, MO) and coverslipped with Fluoromount G™ (Southern Biotech, Birmingham, AL). Finally, we captured fluorescence images using an Olympus confocal system.

To investigate the axons projecting from GABAergic CeA neurons and PSTN CR neurons to the HMNs, sections containing the 12N were washed in PBST and incubated with a rabbit polyclonal anti-mCherry primary antibody (1:5000, cat. #632496, Clontech, San Diego, CA) for 24 h at 4 °C. For chromogenic detection of mCherry, sections were then washed three times in PBS (5 min each) and incubated in donkey anti-rabbit biotinylated IgG (1:1000, cat. #711-065-152, Jackson ImmunoResearch, West Grove, PA) in PBST for 2 h at RT. The sections were then washed in PBS and incubated with an avidin–biotin–peroxidase complex (1:1000, cat. #PK-6100, Vector Laboratories, Burlingame, CA) in PBST for 2 h at RT. Then, the sections were washed and incubated in a solution of 3, 3′ -diaminobenzidine (0.2 mg/mL) and 0.005% H_2_O_2_ in PBS until mCherry-immunoreactive axons could be identified. Finally, we placed the immunostained sections on the glass slides, dehydrated them, and cover-slipped them as described previously [25].

### Imaging and Data Analysis

For the whole-brain mapping of monosynaptic inputs, images of sections were captured with a 10 × objective on an Olympus microscope (VS-120, Tokyo, Japan) and further imaging analyses were done using Olympus analysis software and ImageJ. The numbers of DsRed-labeled cells (excluding the injection site) were counted automatically by ImageJ. By adjusting the threshold of the image, we set a minimum size of cells in the section as the threshold, and the algorithm automatically counted cells larger than this value. The boundaries of brain nuclei were defined according to the atlas of Paxinos and Franklin [26]. The proportion of inputs from each of the 53 brain regions was calculated as the ratio of the number of afferent cells in each nucleus to the total number of DsRed-labeled cells. In addition, we used a 100 × oil objective on the Olympus microscope to determine whether there were axons in the 12N from the CeA and PSTN. We also used an Olympus confocal system to calculate the co-labeling rates of GABAergic and CR neurons occupying the total DsRed-labeled populations in the CeA and PSTN, respectively. Lastly, we analyzed the proportion of inputs from each of the 53 brain regions [26] and the co-labeling rates of GABAergic and CR neurons in the total DsRed-labeled populations (*n* = 4). We used GraphPad prism 7.0 and the paired *t* test for statistical analysis. All data are presented as the mean ± SEM (standard error of the mean).

## Results

### Identification of Monosynaptic Inputs to HMNs Using a Rabies-Based System

**Fig. 1:**
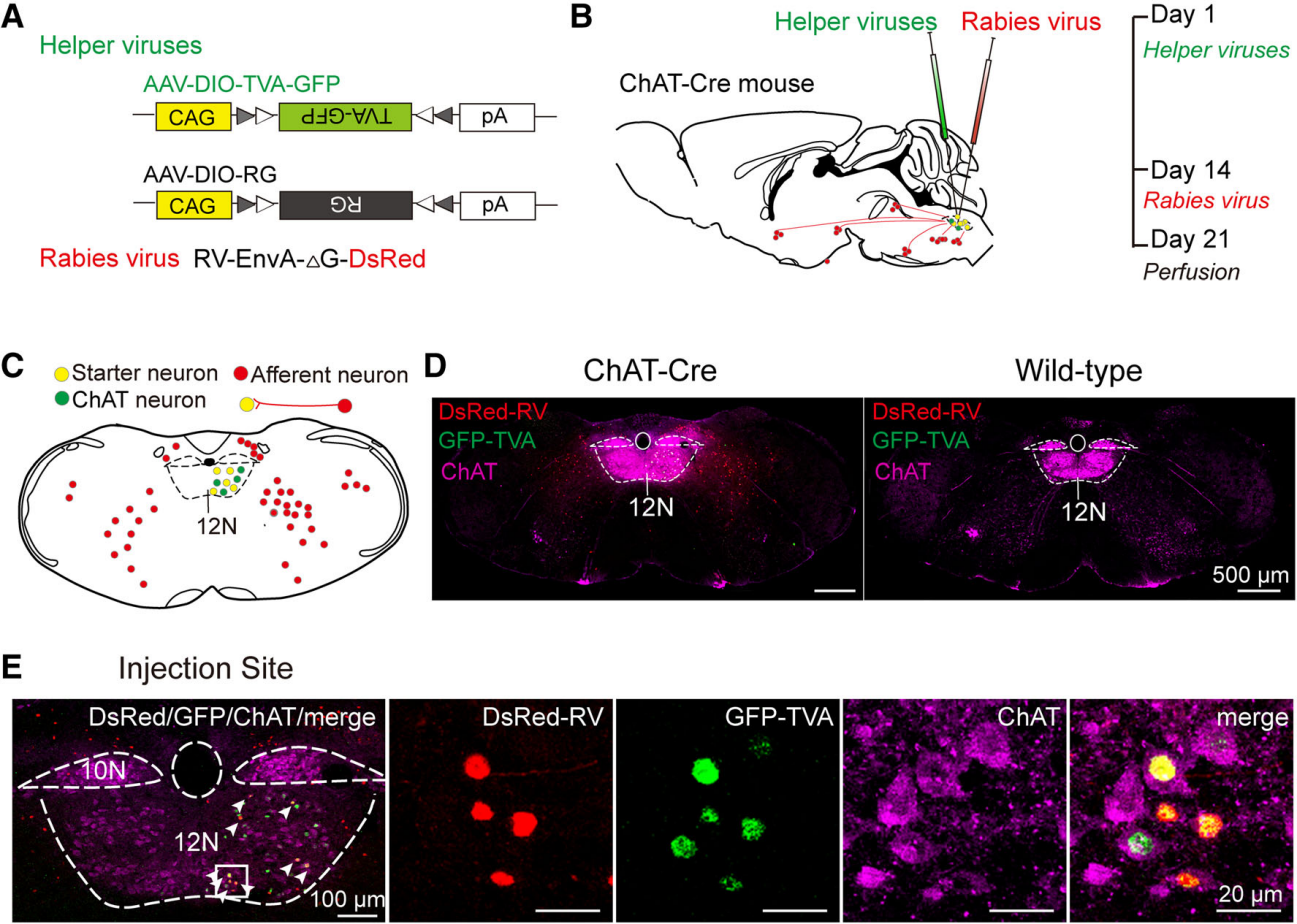
Experimental system based on trans-synaptic rabies virus tracing to identify monosynaptic inputs to HMNs. **A** Design of viral vectors for rabies virus-based trans-synaptic retrograde tracing, including helper viruses with Cre-dependent expression of TVA receptor (AAV-CAG-DIO-TVA-GFP) and RG (AAV-CAG-DIO-RG). The rabies virus was genetically modified by pseudotyping with EnvA (RV-EvnA-DsRed). **B** Schematic of the 12N injection procedure and experimental timeline for helper viruses and rabies virus in the ChAT-Cre mouse. **C** Schematic coronal section to illustrate viral infection in 12N (yellow, starter neurons; green, AAV helper virus-infected neurons; red, rabies virus-infected neurons). **D** Representative images showing rabies virus-labeled neurons in a ChAT-Cre mouse (left) but not in a wild-type mouse (right) (scale bars, 500 µm). **E** A representative section stained with ChAT (purple, left). The section was infected with helper virus (green) and rabies virus (red); starter neurons are restricted to the unilateral 12N (right four panels; scale bar, 20 µm). 10N, vagus nerve nucleus; 12N, hypoglossal nucleus.

To identify the monosynaptic inputs to HMNs, we applied a trans-synaptic viral system based on a modified rabies virus [16] using a transgenic mouse line expressing Cre recombinase in cholinergic neurons (ChAT-Cre mice) [17, 24]. We first injected two Cre-dependent helper viruses (AAV-CAG-DIO-TVA-GFP and AAV-CAG-DIO-RG) to express the avian receptor (TVA protein) and the rabies glycoprotein G (RG) in the unilateral 12N (Fig. 1A, B, Fig. S1). Two weeks later, the modified rabies virus (RV-EnvA-ΔG-DsRed) was injected into the same site (Fig. 1A, B). This rabies virus only infected neurons with TVA expression and spread retrogradely with the RG expression in the brain (Fig. 1C). After seven days, the starter neurons were defined and characterized by the co-expression of DsRed and GFP; they were restricted to the 12N ipsilateral to the injection site (Fig. 1E). Moreover, we observed DsRed-labeled neurons in other regions around the 12N, which represented the monosynaptic afferents to cholinergic HMNs (Fig. 1C–E). The same strategy was used in wild-type mice, and we did not detect any DsRed-positive neuron anywhere in the brains of these control mice (Fig. 1D). Therefore, this technique is reliable for visualizing monosynaptic afferent inputs to HMNs from the whole brain.

### Whole-Brain Inputs to HMNs

**Fig. 2:**
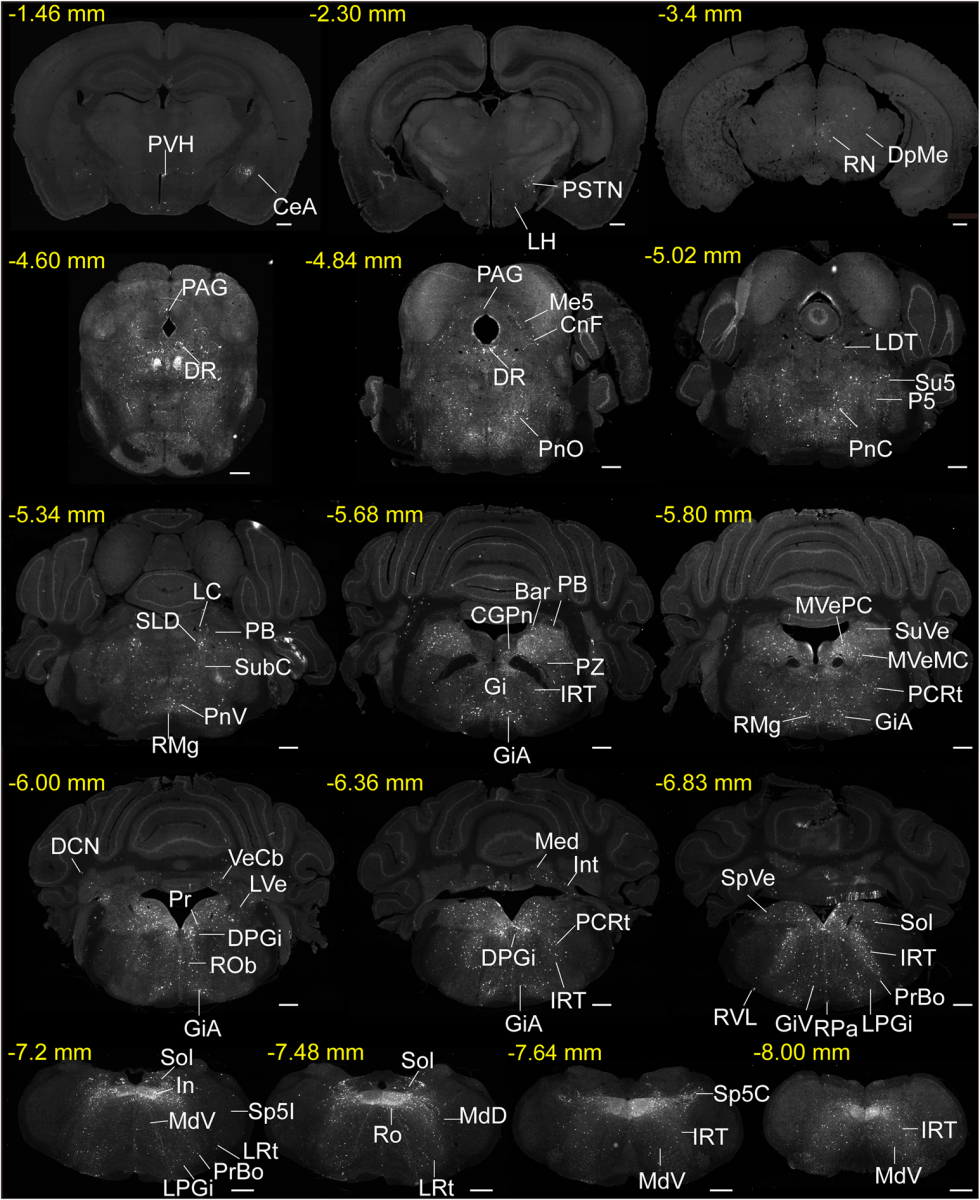
Representative images of monosynaptic inputs to HMNs from the whole brain. Regions are labeled according to the mouse brain atlas [26] (scale bars, 500 μm). Abbreviations: CeA, central amygdaloid nucleus; PVH, paraventricular hypothalamus; LH, lateral hypothalamic area; PSTN, parasubthalamic nucleus; DpMe, deep mesencephalic nucleus; RN, red nucleus; PAG, periaqueductal gray; DR, dorsal raphe nucleus; Me5, mesencephalic trigeminal nucleus; CnF, cuneiform nucleus; PnO, pontine reticular nucleus, oral part; PnC, pontine reticular nucleus, caudal part; LDT, laterodorsal tegmental nucleus; P5, peritrigeminal zone; Su5, supratrigeminal nucleus; LC, locus coeruleus; SLD, sublateral dorsal nucleus; PB, parabrachial nucleus; PnV, pontine reticular nucleus, ventral part; SubC, subcoeruleus nucleus; RMg, raphe magnus nucleus; Gi, gigantocellular reticular nucleus; GiA, gigantocellular reticular nucleus, alpha part; CGPn, central gray of the pons; Bar, Barrington’s nucleus; PZ, parafacial zone; IRT, intermediate reticular nucleus; PCRt, parvocellular reticular nucleus; MVeMC, medial vestibular nucleus, magnocellular part; MVePC, medial vestibular nucleus, parvocellular part; SuVe, superior vestibular nucleus; LVe, lateral vestibular nucleus; DCN, deep cerebellar nucleus; VeCb, vestibulocerebellar nucleus; Pr, prepositus nucleus; DPGi, dorsal paragigantocellular nucleus; ROb, raphe obscurus nucleus; Med, medial (fastigial) cerebellar nucleus; Int, interposed cerebellar nucleus; SpVe, spinal vestibular nucleus; RVL, rostroventrolateral reticular nucleus; RPa, raphe pallidus nucleus; LPGi, lateral paragigantocellular nucleus; GiV, gigantocellular reticular nucleus, ventral part; Sol, nucleus of the solitary tract; PrBo, pre-Botzinger complex; In, intercalated nucleus of the medulla; LRt, lateral reticular nucleus; Sp5I, spinal trigeminal nucleus, interpolar part; Sp5C, spinal trigeminal nucleus, caudal part; Ro, nucleus of Roller; MdD, medullary reticular nucleus, dorsal part; MdV, medullary reticular nucleus, ventral part.

**Fig. 3:**
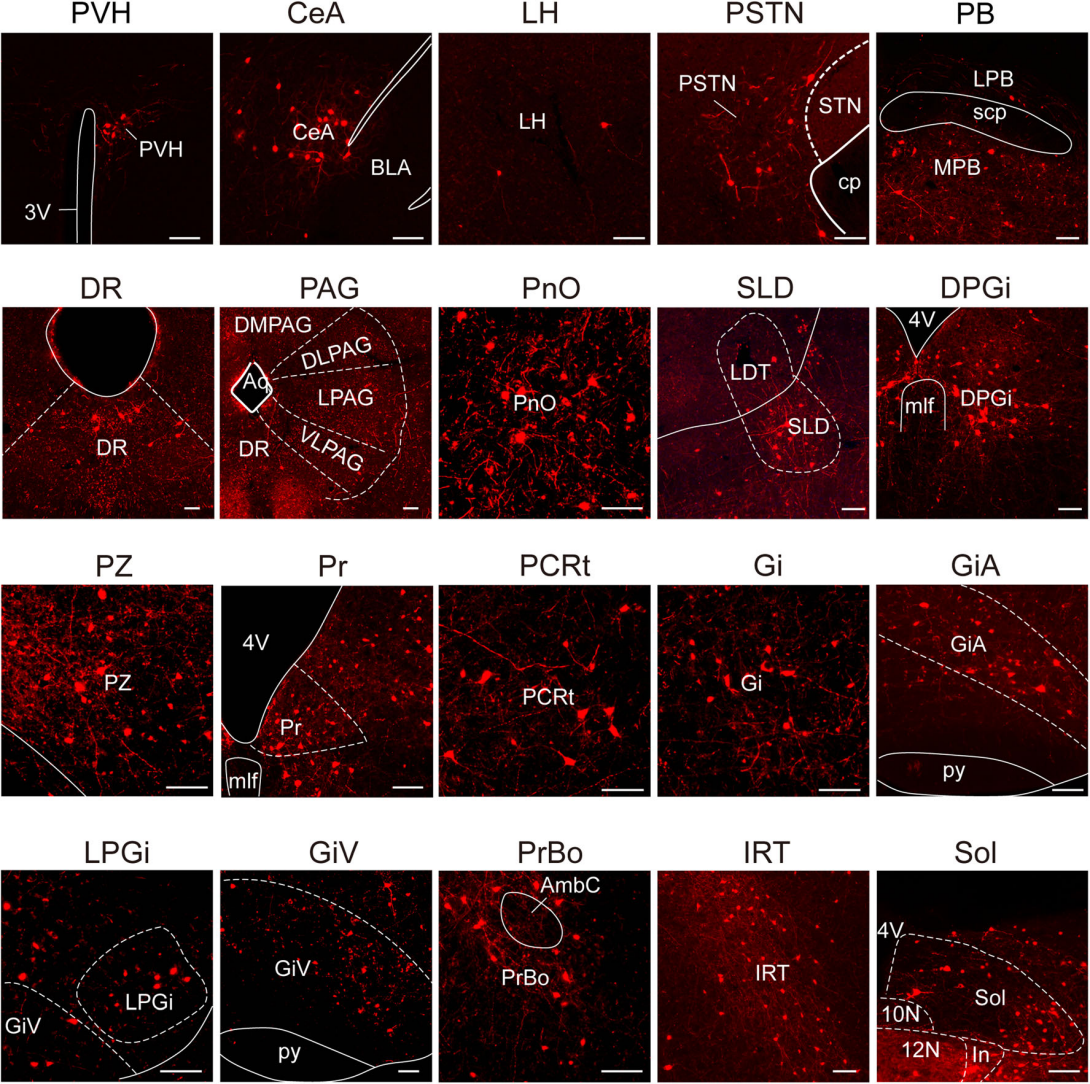
Representative nuclei with monosynaptic inputs to HMNs. Primary inputs to HMNs include multiple nuclei associated with respiration (e.g., Sol, PrBo, PB, ventral medulla, and hypothalamus) and sleep–wake regulation (e.g., PZ, PB, hypothalamus, SLD, and ventral medulla) (scale bars, 100 μm). Abbreviations: PVH, paraventricular hypothalamus; 3V, third ventricle; CeA, central amygdaloid nucleus; BLA, basolateral amygdaloid nucleus, anterior part; LH, lateral hypothalamic area; PSTN, parasubthalamic nucleus; STN, subthalamic nucleus; cp, cerebral peduncle; PB, parabrachial nucleus; LPB, lateral parabrachial nucleus; MPB, medial parabrachial nucleus; scp, superior cerebellar peduncle; DR, dorsal raphe nucleus; PAG, periaqueductal gray; DMPAG, dorsomedial periaqueductal gray; DLPAG, dorsolateral periaqueductal gray; LPAG, lateral periaqueductal gray; VLPAG, ventrolateral periaqueductal gray; Aq, aqueduct; PnO, pontine reticular nucleus, oral part; SLD, sublateral dorsal nucleus; LDT, laterodorsal tegmental nucleus; DPGi, dorsal paragigantocellular nucleus; 4V, fourth ventricle; mlf, medial longitudinal fasciculus; PZ, parafacial zone; Pr, prepositus nucleus; PCRt, parvocellular reticular nucleus; Gi, gigantocellular reticular nucleus; ROb, raphe obscurus nucleus; GiA, gigantocellular reticular nucleus, alpha part; py, pyramidal tract; LPGi, lateral paragigantocellular nucleus; GiV, gigantocellular reticular nucleus, ventral part; PrBo, pre-Botzinger complex; AmbC, ambiguus nucleus, compact part; IRT, intermediate reticular nucleus; 10N, vagus nerve nucleus; 12N, hypoglossal nucleus; In, intercalated nucleus; Sol, nucleus of the solitary tract.

To investigate the inputs to HMNs from the whole brain, we cut serial coronal sections (Fig. 2) after enough infection time for the three injected viruses. Sections from a typical ChAT-Cre brain (Fig. 2) revealed that DsRed-labeled afferent neurons were largely located in the medulla oblongata, while some were found in the pons and midbrain. Furthermore, a few input neurons were found in the hypothalamus, amygdala, and cerebellum (Fig. 2). To display the whole-brain distribution of neurons presynaptic to HMNs in detail (Fig. 3), we enlarged and selected representative coronal images from the following major afferent nuclei: the paraventricular hypothalamus (PVH), CeA, lateral hypothalamus (LH), PSTN, parabrachial nucleus (PB), dorsal raphe nucleus (DR), periaqueductal gray (PAG), pontine reticular nucleus, oral part (PnO), sublaterodorsal tegmental nucleus (SLD), dorsal paragigantocellular nucleus, parafacial zone (PZ), prepositus nucleus, parvocellular reticular nucleus (PCRt), gigantocellular reticular nucleus (Gi), gigantocellular reticular nucleus, alpha part (GiA), lateral paragigantocellular nucleus (LPGi), gigantocellular reticular nucleus, ventral part (GiV), pre-Botzinger complex (PrBo), intermediate reticular nucleus (IRT), and Sol.

**Fig. 4:**
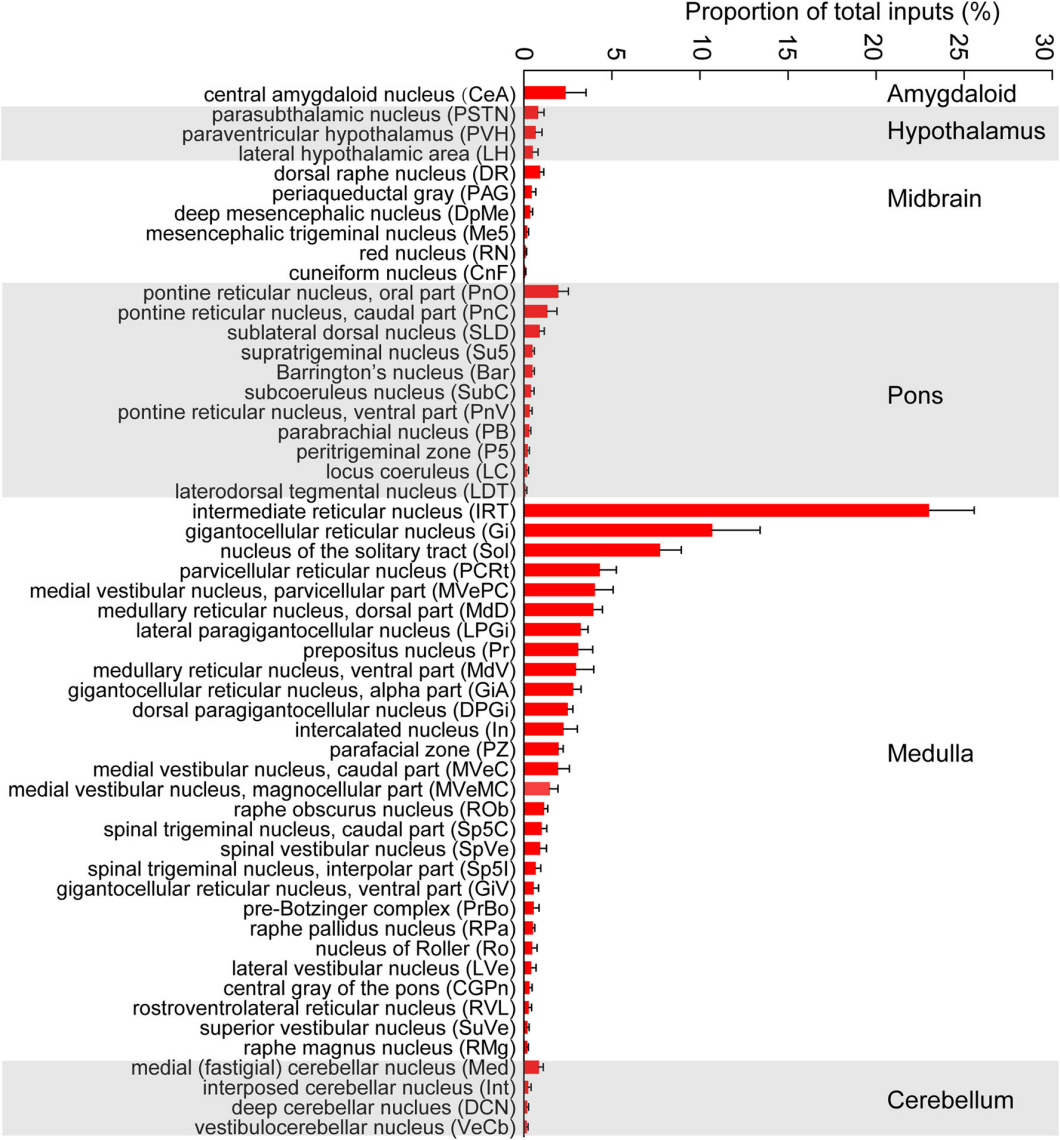
Statistical analysis of the whole-brain distribution of monosynaptic inputs to HMNs. Average proportion of DsRed-labeled neurons in 53 brain regions with > 0.1% of the total input to HMNs in ChAT-Cre mice (*n* = 4). Brain areas are grouped into six general structures: the amygdala, hypothalamus, midbrain, pons, medulla, and cerebellum.

Next, we conducted a statistical analysis of the distribution of input nuclei from the whole brain based on the ratio of the number of DsRed-labeled neurons in each nucleus to the total number of labeled neurons in each brain (Fig. 4, *n* = 4). We identified 53 nuclei that each had a ratio of > 0.1% of the total number of labeled neurons (Fig. 4, *n* = 4). The inputs to HMNs originated in six brain structures: the amygdala, hypothalamus, midbrain, pons, medulla, and cerebellum. Large numbers of neurons providing direct projections to HMNs were found in the IRT (23.02% ± 2.55%), Sol (10.07% ± 2.71%), and Gi (7.74% ± 1.21%) of the medulla. The ventral medullary reticular region, including the LPGi (3.25% ± 0.40%), GiA (2.82% ± 0.44%), and GiV (0.57% ± 0.26%), also had strong projections to HMNs. The PnO (1.96% ± 0.58%) in the pons, and the CeA (2.38% ± 1.15%), were the other major nuclei providing inputs to HMNs.

Our results showed that HMNs received mass monosynaptic inputs from regions associated with respiration, such as the Sol, PrBo (0.58% ± 0.28%), ventral medulla (VM), PB (0.32% ± 0.09%), and hypothalamus. Some nuclei controlling sleep and wakefulness, including the PZ (1.99% ± 0.25%), SLD (0.91% ± 0.25%), PB (0.32% ± 0.09%), DR (0.94% ± 0.20%), PAG (0.46% ± 0.22%), PSTN (0.82% ± 0.32%), and LH (0.51% ± 0.30%), also projected direct inputs to HMNs (Fig. 4, *n* = 4). In summary, our results provide a concrete input distribution in the whole brain and identify specific regions that are known to be involved in the regulation of different physiological functions.

### HMNs Receive Inputs from GABAergic CeA Neurons

**Fig. 5:**
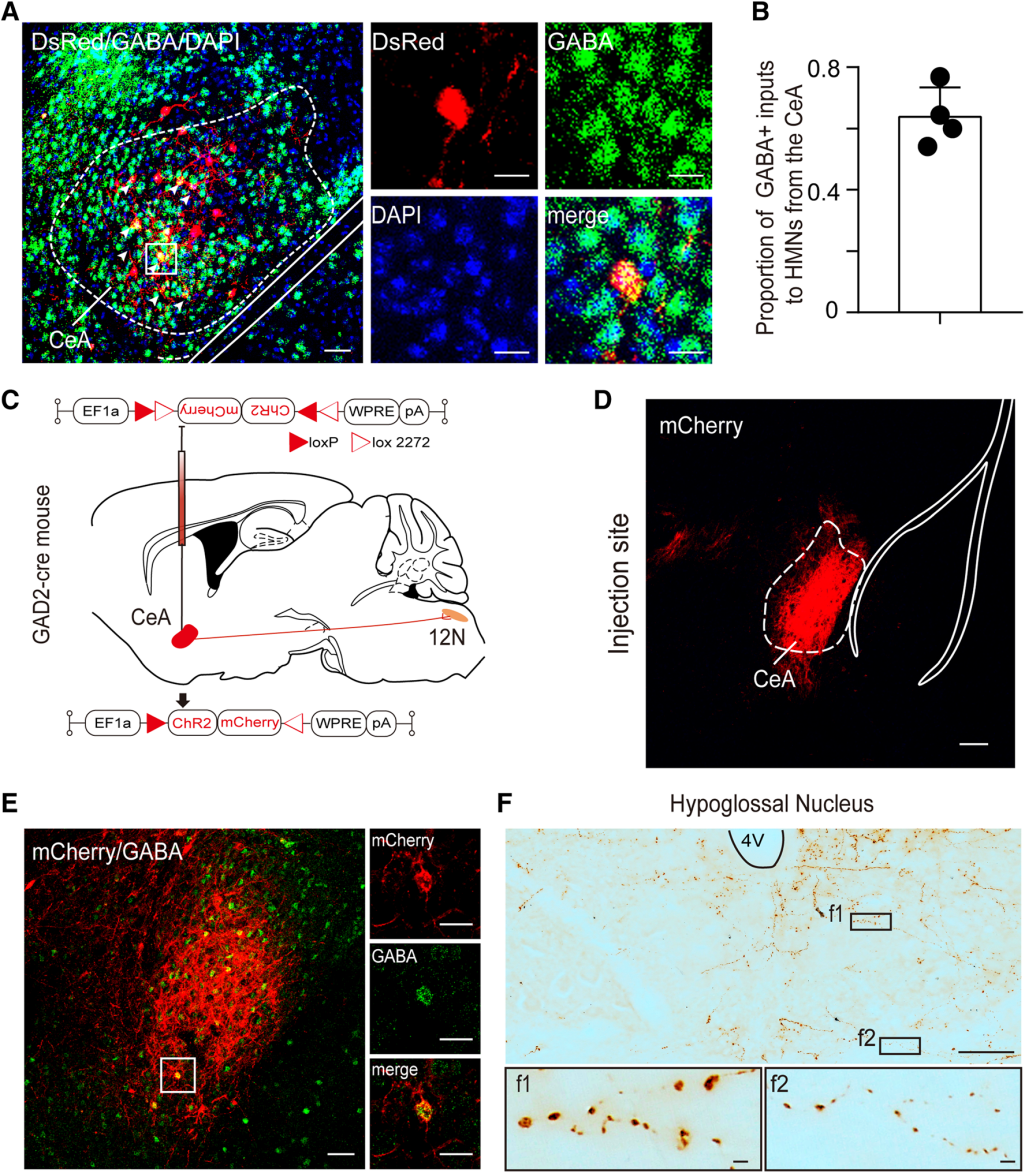
Cholinergic HMNs receive monosynaptic inputs from GABAergic CeA neurons. **A** Left, a large portion of DsRed neurons co-localize with GABA in the CeA (scale bar, 50 μm). Right, higher magnification images of the area outlined by the white box (scale bars, 10 µm). **B** The co-labeling rate of GABAergic neurons was 63.87% ± 4.83% of the total number of DsRed-labeled neurons in the CeA. **C** Schematic of the CeA injection site and viral vectors for AAV-EF1a-DIO-ChR2-mCherry in GAD2-Cre mice. **D** Representative image of the location of viral AAV-EF1a-DIO-ChR2-mCherry (red) infection covering most of the CeA (scale bar, 100 μm). **E** Left, fluorescence image showing that the neurons infected with ChR2-mCherry are mostly co-localized with GABA in the CeA (scale bar, 50 μm). Right, higher magnification images of the area outlined by the white box (scale bars, 20 µm). **F** Upper, representative image showing mCherry-labeled axons of GABAergic CeA neurons in 12N (scale bar, 100 µm). Lower, higher magnification images of the areas enclosed by the black boxes f1 and f2 (scale bars, 100 μm). CeA, central amygdaloid nucleus.

The CeA is a structure critical for learning, memory, consolidation of fear conditioning, predatory hunting, cataplexy, and emotion [27–31]. A previous study has shown that the CeA has connections with tongue premotor neurons, as demonstrated by retrograde trans-synaptic transport of pseudorabies virus inoculated into the GG [32]. Unexpectedly, we found that HMNs received inputs from the bilateral CeA, as shown by our retrograde rabies-based system. Moreover, the ipsilateral CeA tended to have more DsRed-labeled neurons than that on the contralateral side (1.66% ± 0.76% *versus* 0.88% ± 0.33%, *n* = 4, Fig. S3). The DsRed-labeled neurons in the CeA were mainly co-localized with GABA (63.87% ± 4.83%; Fig. 5A, B). To further confirm that GABAergic CeA neurons send direct projections to 12N, we injected an AAV expressing Cre-dependent ChR2-mCherry into the CeA of GAD2-Cre mice (Fig. 5C, D). After AAV injection, the mCherry-expressing neurons in the CeA mainly overlapped with GABA (Fig. 5E, Fig. S2). In addition to finding funicular mCherry-labeled axons from the CeA in the bilateral 12N, we also observed mCherry axons with varicosities and boutons in 12N (Fig. 5F), indicating that the GABAergic CeA neurons project directly to 12N. Thus, our results from retrograde and anterograde tracing demonstrated that the HMNs receive direct innervation from GABAergic CeA neurons.

### HMNs Receive Inputs from Calretinin Neurons in the PSTN

**Fig. 6:**
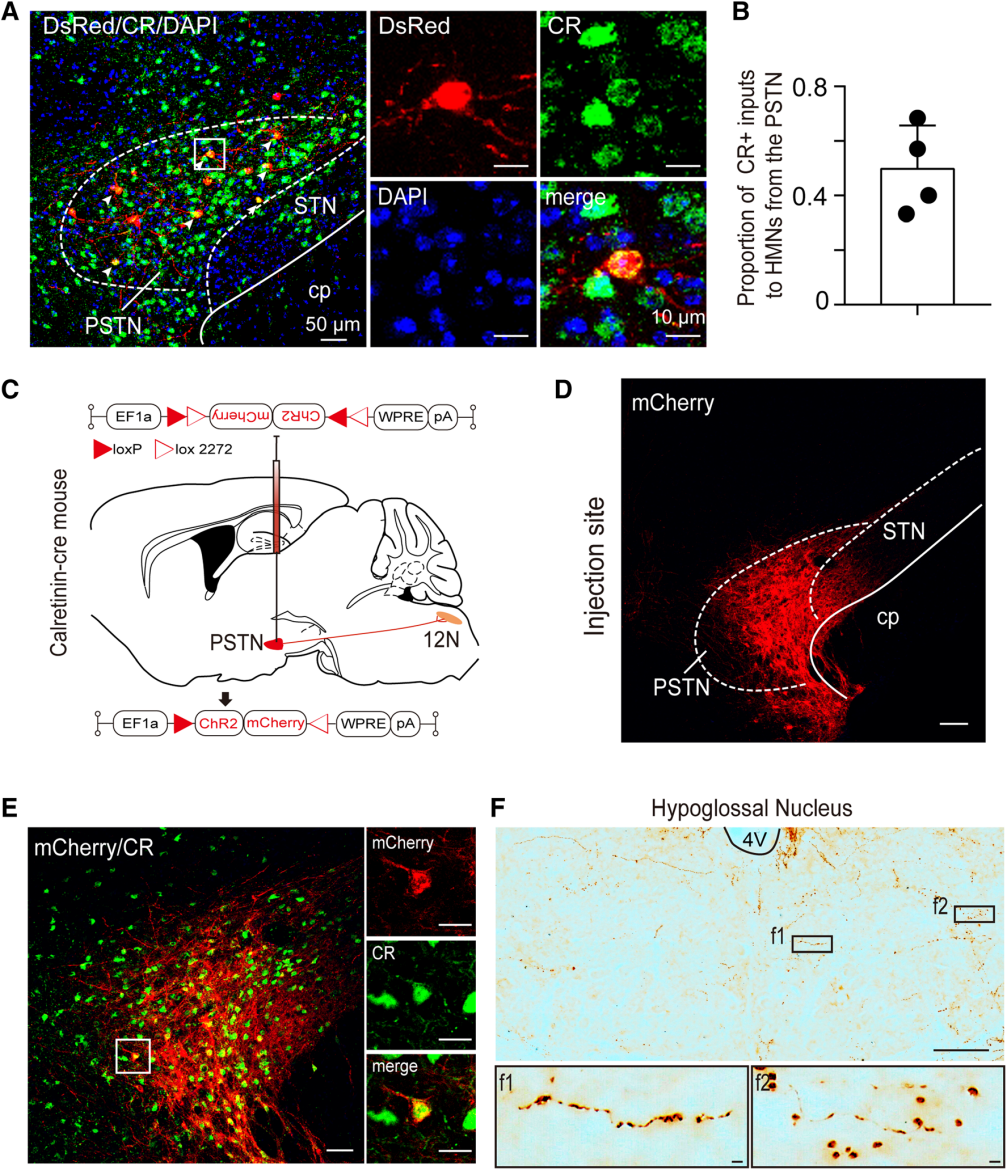
Cholinergic HMNs receive monosynaptic inputs from PSTN calretinin neurons. **A** Left, image showing that DsRed-labeled neurons are highly co-localized with CR neurons in the PSTN (scale bar, 50 μm). Right, higher magnification images of the white boxed area (scale bars, 10 µm). **B** The co-labeling rate of CR neurons was 49.72% ± 8.00% of the total number of DsRed-labeled neurons in the PSTN. **C** Schematic of the PSTN injection site and viral vectors for AAV-EF1a-DIO-ChR2-mCherry in CR-Cre mice. **D** Representative image showing that the location of viral AAV-EF1a-DIO-ChR2-mCherry (red) infection covered most of the PSTN (scale bar, 100 μm). **E** Left, representative fluorescence image showing that almost all the neurons infected with ChR2-mCherry co-localized with CR in the PSTN (scale bar, 50 μm). Right, higher magnification images of the area outlined by the white box (scale bars, 20 µm). **F** Upper, representative image showing mCherry-labeled axons of PSTN CR neurons in the 12N (scale bar, 100 µm). Lower, higher magnification images of the areas outlined by the black boxes f1 and f2 (scale bars, 100 μm). CR, calretinin; cp, cerebral peduncle; STN, subthalamic nucleus; PSTN, parasubthalamic nucleus.

The hypothalamus is a heterogeneous structure that participates in the regulation of various functions, including sleep–wake behavior, feeding, energy balance, and reproductive behaviors [33–35]. The PSTN has recently been identified as a small subregion in the hypothalamus that is crucial in sleep–wake and feeding behavior [36, 37]. Here, we found that HMNs received small afferent inputs from the bilateral PSTN (Fig. 2), and the ipsilateral PSTN tended to have more projections than the contralateral PSTN (0.61% ± 0.23% *vs* 0.29% ± 0.07%, *n* = 4, Fig. S3). As demonstrated by immunofluorescent staining, DsRed-labeled neurons in the PSTN were co-labeled with CR-immunoreactive labeling (49.72% ± 8.00%; Fig. 6A, B). Furthermore, we injected an anterograde-tracing virus expressing Cre-dependent ChR2-mCherry into the PSTN in CR-Cre mice to confirm that PSTN CR neurons sent direct projections to the 12N (Fig. 6C–E, Fig. S2). After three weeks, we observed funicular mCherry-labeled axon terminals in the bilateral 12N, as well as many varicosities and boutons expressing mCherry in the 12N (Fig. 6F). These results indicated that PSTN CR neurons directly project to the 12N and that PSTN CR neurons form connections with ChAT neurons within the 12N.

## Discussion

To better understand the pathogenesis of diseases caused by HMN dysfunction, it is necessary to elucidate the neuromodulatory mechanisms that influence them. Some studies have reported afferent inputs to 12N using traditional retrograde-tracing techniques, including horseradish peroxidase and pseudorabies virus [12, 14]. Due to the non-specificity and inefficiency of these tracers, the data likely do not represent the comprehensive monosynaptic inputs to the cholinergic neurons of the 12N [14]. In the present study, we used a rabies-based system to comprehensively and accurately label monosynaptic inputs to HMNs at the whole-brain scale and found that HMNs received extensive direct inputs from the brainstem. Moreover, we found monosynaptic inputs from the CeA, PSTN, LH, and PVH. Taken together, our results provide a comprehensive and precise map of the spatial pattern of presynaptic inputs that modulate the activity of cholinergic HMNs for different behaviors.

### Comparison with Previous Retrograde Tracing Studies

The afferent inputs to HMNs have been extensively investigated for their critical roles in the modulation of tongue movements. Previous studies using classical non-specific tracers have shown that projections to the 12N originate from the brainstem reticular formation, the sensory trigeminal complex, and the Sol [14]. Injection of multi-synaptic pseudorabies virus into tongue muscles has revealed many multi-synaptic connections with HMNs in the brainstem [12, 15]. However, the non-specificity and multi-synaptic delivery characteristics of pseudorabies viruses make these results inaccurate. Therefore, a recent study used the ChAT-Cre: Rabies-G-crossed mouse line to specifically express glycoprotein G in cholinergic neurons and injected a glycoprotein G-defective rabies virus into the GG to label the premotor neurons of the HMNs innervating that muscle [38]. Although this approach achieved monosynaptic retrograde tracing with improved transfer efficiency, this tracing system was still defective for its spurious two-step labeling if any premotor neurons expressed ChAT, which may have induced possible deviations in the tracing results [38]. It is unclear whether the premotor neurons of HMNs express ChAT, although a previous study reported that some cholinergic neurons in the IRT of the brainstem project to HMNs [39], and there may be other hypoglossal premotor neurons that express ChAT. Meanwhile, that study used the ChAT-Cre: Rabies-G-crossed mouse line [38] and focused on whether the premotor circuitry of jaw and tongue motoneurons contains elements for coordination, rather than investigating the primary input circuitry of HMNs from the whole brain [38]. In addition, no previous studies have quantified the number of nuclei that input to HMNs, and most studies did not focus on HMN inputs from forebrain regions. Here, we used a rabies virus-mediated retrograde tracing system in ChAT-Cre mice, which allowed for specific labeling of whole-brain monosynaptic inputs to HMNs. We built a detailed and quantitative map of these inputs by counting the proportion of inputs from each region and found that the majority of these inputs originated in the IRT, Gi, and Sol of the medulla, which is consistent with previous results [12, 14, 40]. In addition, the PCRt, MVePC, MdD, and LPGi in the medulla each contained a large number of labeled cells that innervated HMNs. Moreover, relatively dense inputs to HMNs also originated from the PnO, PnC, and SLD in the pons, as well as the DR and PAG in the midbrain.

Our results not only confirmed previous studies that used conventional retrograde tracing but also yielded the novel finding that HMNs receive direct inputs from the CeA and hypothalamus. The CeA is involved in the expression of conditioned responses to aversive oral stimuli, including the gaping and tongue protrusion that are driven by central pattern generators and other premotor neurons in the ponto-medullary reticular formation [41]. A previous study showed that the CeA is the only amygdalar nucleus to send axons to the pons and medulla and that it has serial connections with premotor neurons of the tongue musculature [32]. Here, we found that GABAergic CeA neurons directly innervated HMNs, which may explain the changes of tongue muscle activity in response to aversive oral stimuli. The hypothalamus is a higher-order center of the autonomic nervous system and maintains essential homeostatic processes including respiration, sleep–wake behavior, and feeding. Here, we found primary inputs to HMNs from the hypothalamic PVH, PSTN, and LH, which morphologically corroborates the roles of the hypothalamus in the regulation of tongue activity. The proportion of inputs from the PSTN projecting to HMNs among hypothalamic inputs was relatively dense; ~ 50% co-localized with CR neurons. PSTN CR neurons might engage in the regulation of feeding [37] and modulate the activity of HMNs during eating.

### Neural Circuitry Underlying Modulation of HMN Activity During Respiration

HMNs innervate the GG muscle of the tongue, which plays a significant role in maintaining an open airspace for effective breathing [42]. The Sol, an important medullary area, integrates and relays afferent signals from the hypoglossal nerves to the respiratory center. Previous tracing studies have suggested that the Sol contains neurons that project densely to 12N [14, 40]. Our results support these findings, as we revealed that numerous Sol neurons provided monosynaptic inputs onto HMNs. These inputs could play an important role in the well-established respiratory reactivity of the GG. The PrBo, a compact medullary region, is essential for generating normal breathing rhythms and patterns [43]. Here, we found that the PrBo had sparse axonal terminals on HMNs, indicating that the PrBo–HMN pathway could play a role in maintaining the activity of HMNs in order to maintain an open upper airway.

The hypercapnia, hypoxia, and negative intrapharyngeal-airway pressure created by inspiratory effort against a blocked airway induce progressive activation of the GG. The Sol, PB, and ventrolateral medulla receive projections from chemosensory neurons in the retrotrapezoid nucleus, so they sense the hypercapnia, hypoxia, and other chemosensory information caused by apnea [44]. Our results indicated that all of these regions affect GG activity by innervating HMNs directly. The PVH and LH are critically involved in respiratory control. The most prominent role of the PVH is its involvement in mediation of the respiratory response to hypoxia [45]. Meanwhile, the activity of LH neurons increases in response to hypercapnia [46]. Here, we found that HMNs received direct inputs from the PVH and LH, which helps to explain why GG activity increases quickly in response to hypercapnia and hypoxia.

### Neural Circuitry Underlying the Modulation of HMN Activity During Sleep–Wake Behavior

OSA increases the incidence of cardiovascular diseases such as angina, myocardial infarction, and hypertension and reduces sleep quality to induce excessive daytime sleepiness that can impair work performance [47]. OSA is a state-dependent process ultimately caused by the influence of sleep–wake circuits on pharyngeal muscle tone in individuals with an already narrow upper airway, especially in terms of the influence of neuromodulators acting upon the HMNs that innervate the GG, which is critical to OSA in humans [47]. Exploring the neural mechanisms that regulate HMNs may be critical for the identification and development of new pharmacological strategies to augment GG activity in sleep, especially during REM sleep, as potential treatments for OSA. In the present study, we found that many nuclei involved in sleep–wake regulation sent inputs to HMNs, such as the VM, SLD, PZ, and DR, which might affect HMN activity during sleep–wake transitions.

The VM (including the GiA, GiV, and LPGi) is involved in the regulation of the sleep–wake cycle [48]. It has been reported that GABAergic VM neurons powerfully promote REM sleep and that glutamatergic neurons participate in the regulation of wakefulness [48]. Glutamatergic SLD neurons trigger REM sleep and muscle hypotonia [49] by activating glycinergic/GABAergic premotor neurons that are localized in the VM [50]. Our results revealed that HMNs are one of the major targets of both the VM and SLD, indicating that the activity of HMNs is affected by these nuclei across sleep–wake states. This finding contributes to clarifying the mechanism for lingual muscle tone being at its lowest during REM sleep in OSA patients.

The PZ is located in the medulla oblongata and is lateral and dorsal to the facial nerve. The GABAergic PZ has been identified as a medullary slow-wave sleep-promoting center [51–53], and here we found that the PZ projected directly to HMNs. This may be related to the decreased activity of HMNs during NREM sleep. In addition, we found that other regions known to be involved in sleep–wake regulation such as the PB, PAG, DR, and hypothalamus sent few afferents to HMNs. Hence, these regions may participate in the regulation of HMN activity, along with modulating sleep–wake states.

In summary, we have comprehensively mapped the monosynaptic afferents to HMNs and provide a new perspective for exploring the circuit mechanisms underlying the modulation of HMNs. Unexpectedly, we revealed for the first time that the CeA and the hypothalamus innervate HMNs directly and also provided evidence for other neural pathways that may be involved in the regulation of HMN activity during respiration and different sleep–wake states. Therefore, our data provide an anatomical basis for the treatment of diseases caused by HMN dysfunction.

## Electronic supplementary material

Below is the link to the electronic supplementary material.

Supplementary material 1 (PDF 179 kb)
