# Supplementary material for: Whole-Brain Monosynaptic Inputs to Hypoglossal Motor Neurons in Mice
Source: Neurosci Bull. 2020 Feb 24;36(6):585–97. doi: 10.1007/s12264-020-00468-9 (PMC7270309; doi:10.1007/s12264-020-00468-9)
Supplement: Supplementary file 1 — Supplementary material 1 (PDF 179 kb) [file 12264_2020_468_MOESM1_ESM.pdf]

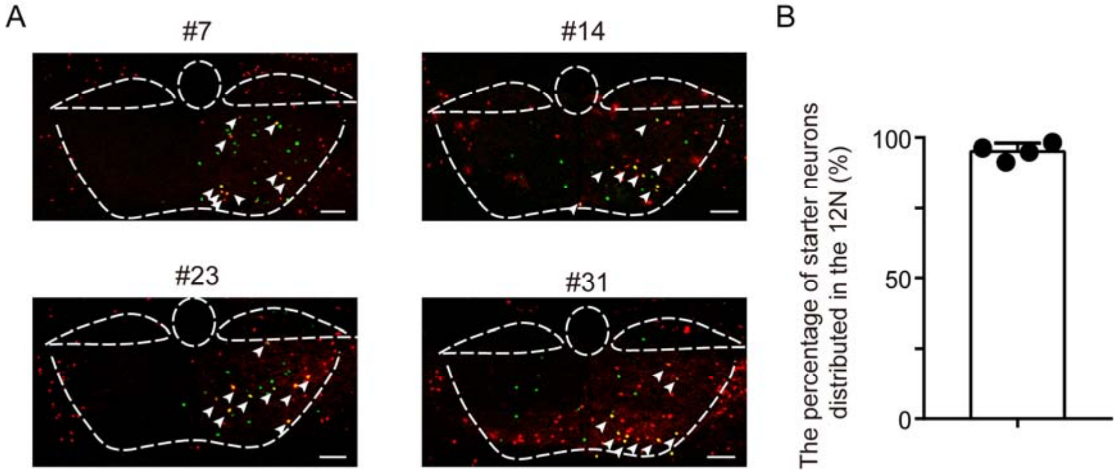

Fig. S1 Distribution and the percentage of starter neurons in the hypoglossal nucleus. **A** Distribution of starter neurons (yellow, marked by arrowheads) in the hypoglossal nucleus of 4 mice (#7, #14, #23, #31) (scale bars, 100 μm). **B** Percentages of starter neurons in the hypoglossal nucleus.

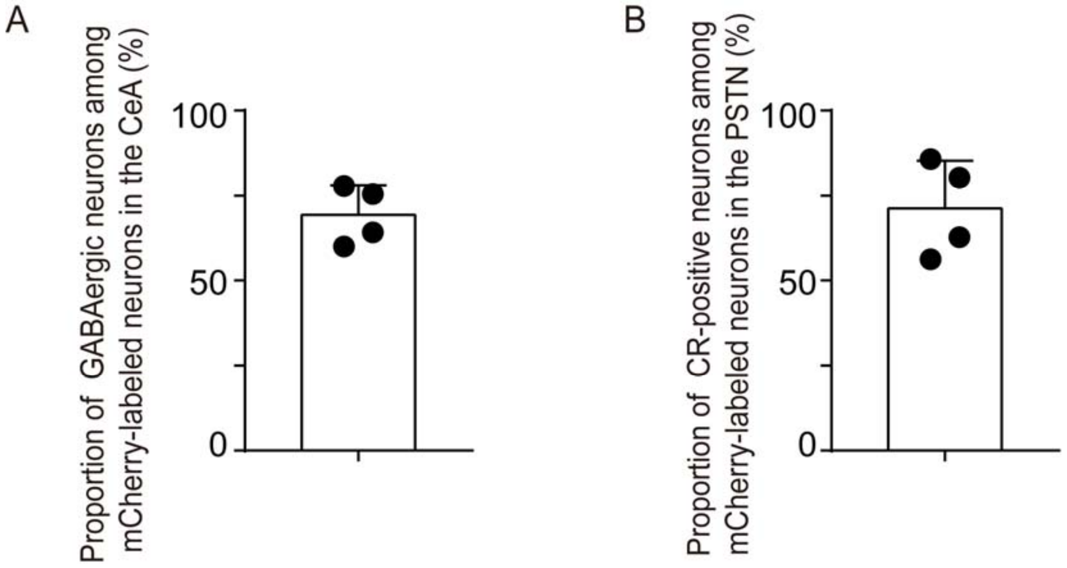

Fig. S2 mCherry-labeled neurons are co-localized with GABAergic neurons in the CeA and with CR neurons in the PSTN. **A** Proportion of GABAergic neurons among mCherry-labeled neurons in the CeA (n = 4). **B** Proportion of CR neurons among mCherry-labeled neurons in the PSTN (n = 4).

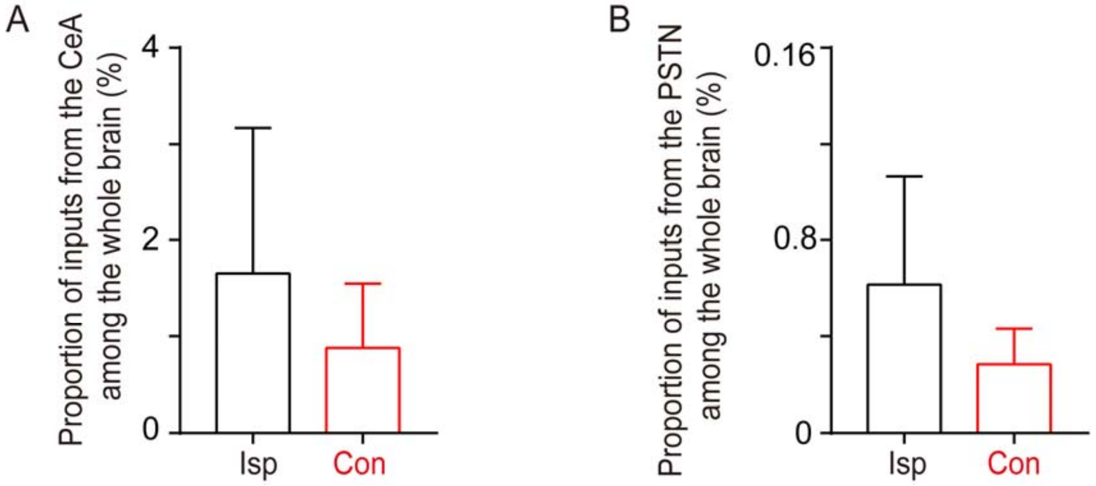

Fig. S3 Ipsilateral CeA and PSTN tend to have more DsRed-labeled neurons than those on the contralateral side. **A** Proportion of inputs from the ipsilateral and contralateral CeA among the whole brain. Data are the mean ± SEM, n = 4, P = 0.1686, paired t-test. **B** Proportion of inputs from the ipsilateral and contralateral PSTN among the whole brain. Data are the mean ± SEM, n = 4, P = 0.1824, paired t-test.
